# Supplementary material for: Immobilized Bisphosphonates as Potential Inhibitors of Bioprosthetic Calcification: Effects on Various Xenogeneic Cardiovascular Tissues
Source: Biomedicines. 2021 Dec 29;10(1):65. doi: 10.3390/biomedicines10010065 (PMC8773418; doi:10.3390/biomedicines10010065)
Supplement: Supplementary file 1 [file biomedicines-10-00065-s001.zip › biomedicines-1512722-supplementary.pdf]

Supplementary materials

IMMOBILIZED BISPHOSPHONATES AS POTENTIAL INHIBITORS OF BIOPROSTHETIC CALCIFICATION: EFFECT ON DIFFERENT XENOGENEIC CARDIOVASCULAR TISSUES

Irina Y. Zhuravleva[1], Anna A. Dokuchaeva[1], Elena V. Karpova[2], Tatyana P. Timchenko[1], Anatoly T.Titov[3], Svetlana S. Shatskaya[4], Yuliya F. Polienko[2]

S1. FTIR-spectroscopy results

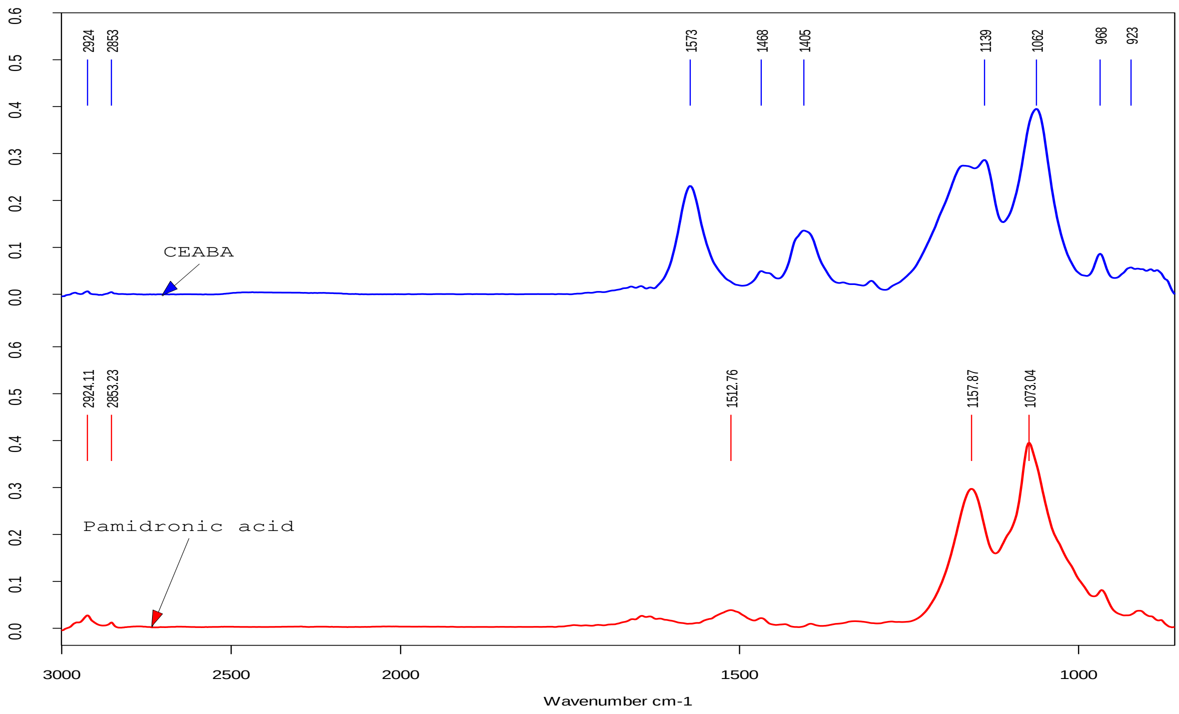

Figure S1. ATR-FTIR spectra of bisphosphonic acids solutions at pH 7

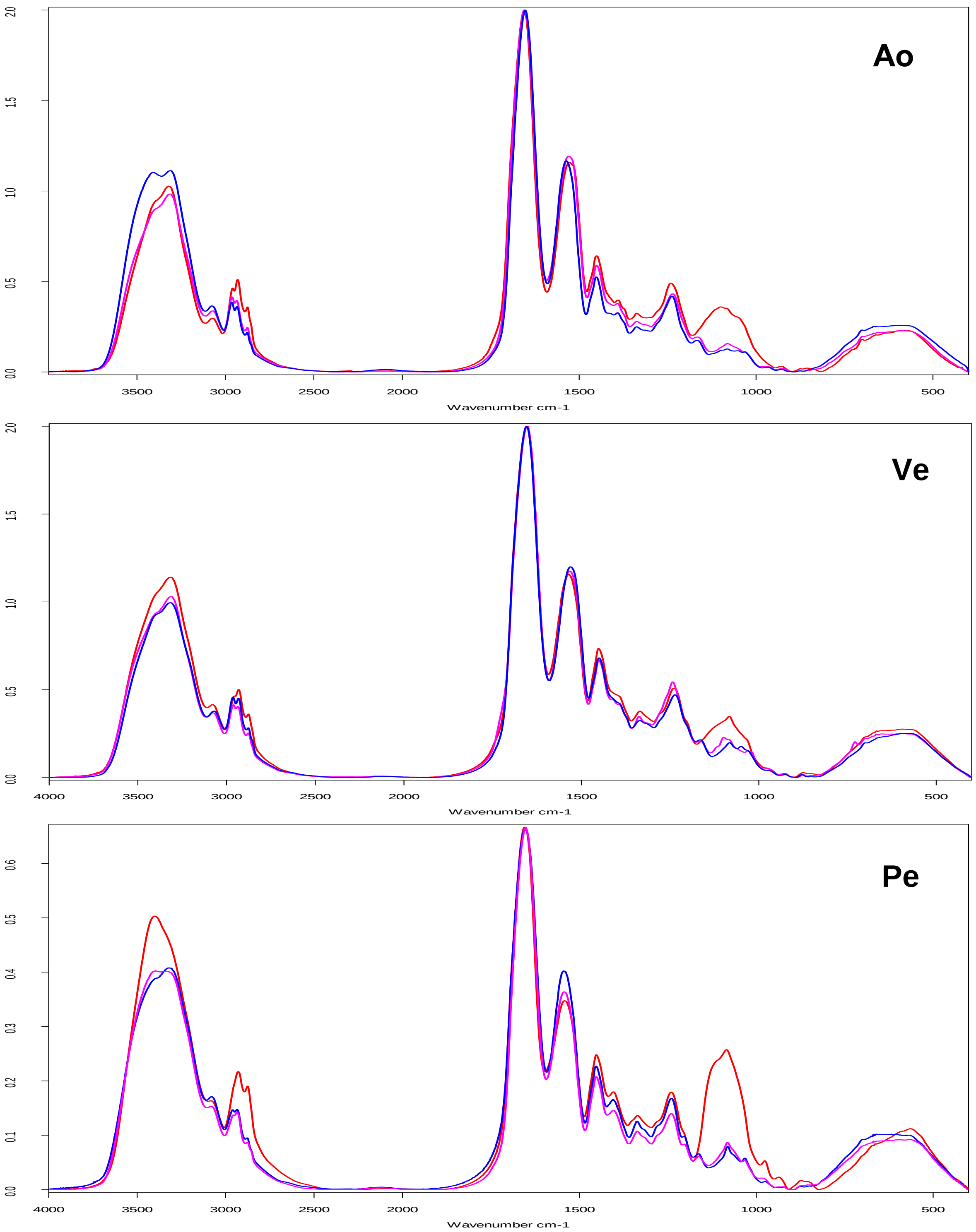

Figure S2. FTIR spectra of bioprosthetic materials: untreated (blue lines), cross-linked with GA (pink lines) or DE (red lines).

S2. SEM of the sample surfaces at different times after the implantation

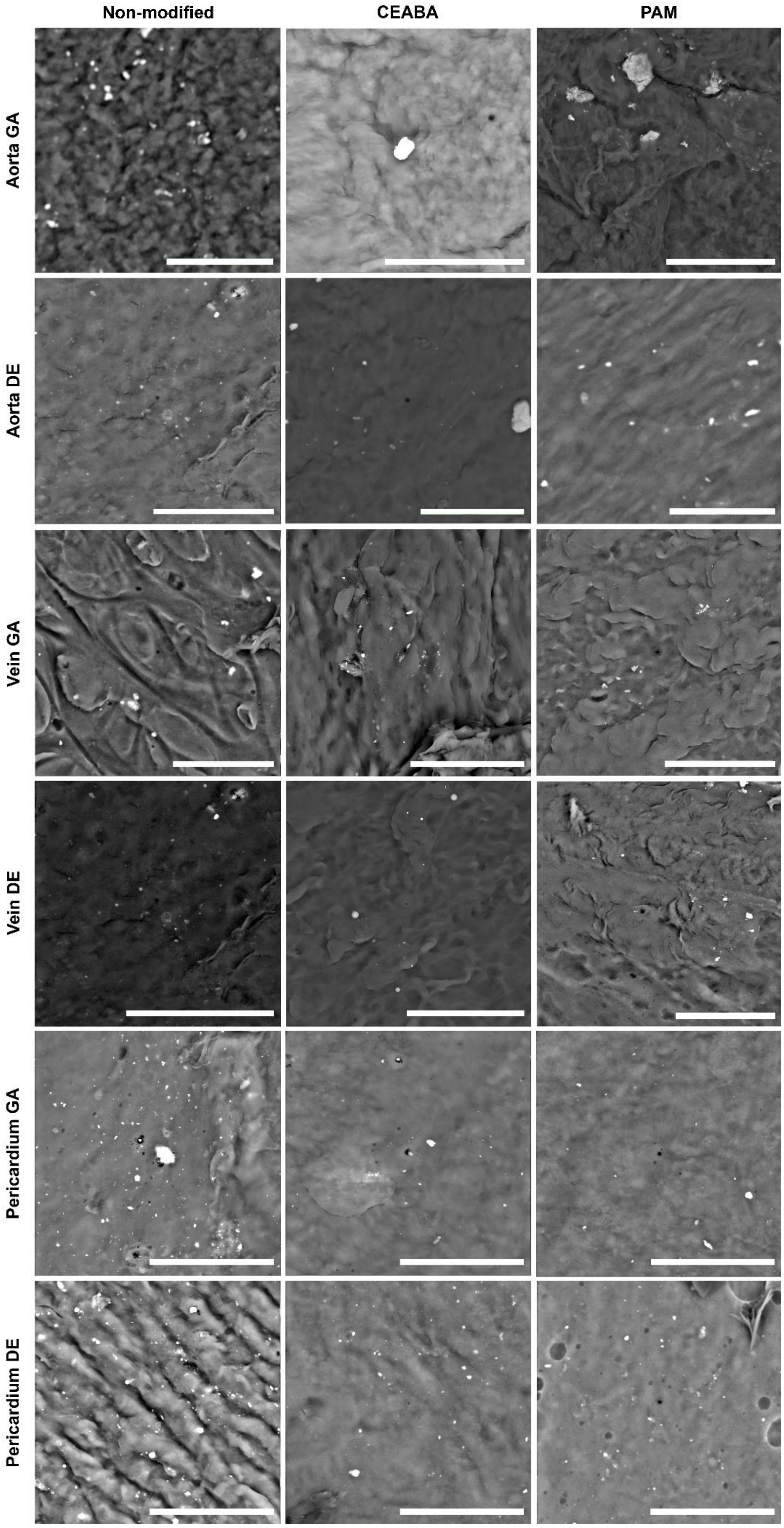

Figure S3. SEM (back-scattering electron) of the bioprosthetic material surfaces in 10 days after subcutaneous implantation in rats. Scale bars 50 μm.

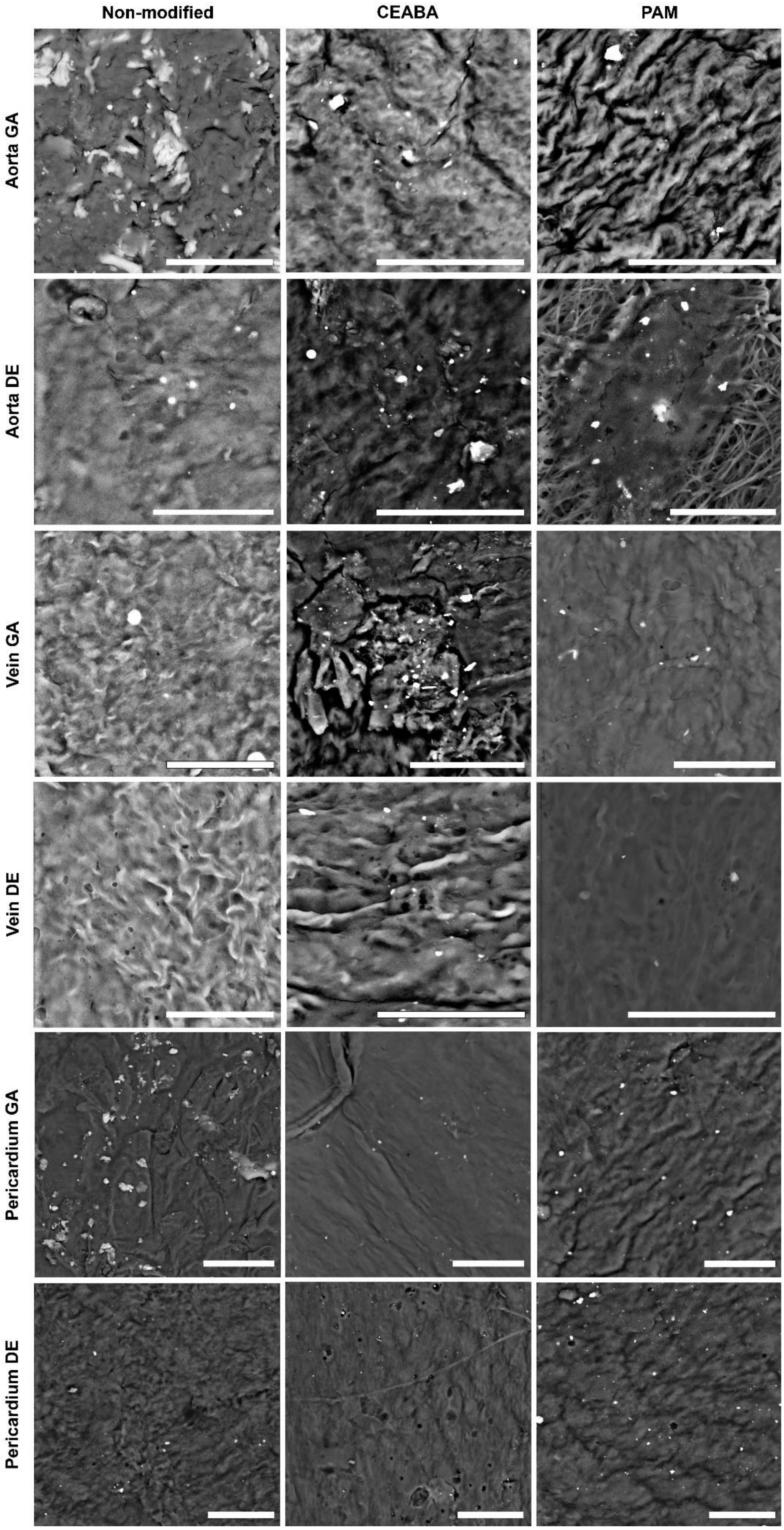

Figure S4. SEM (back-scattering electron) of the bioprosthetic material surfaces in 20 days after subcutaneous implantation in rats. Scale bars 50 μm.

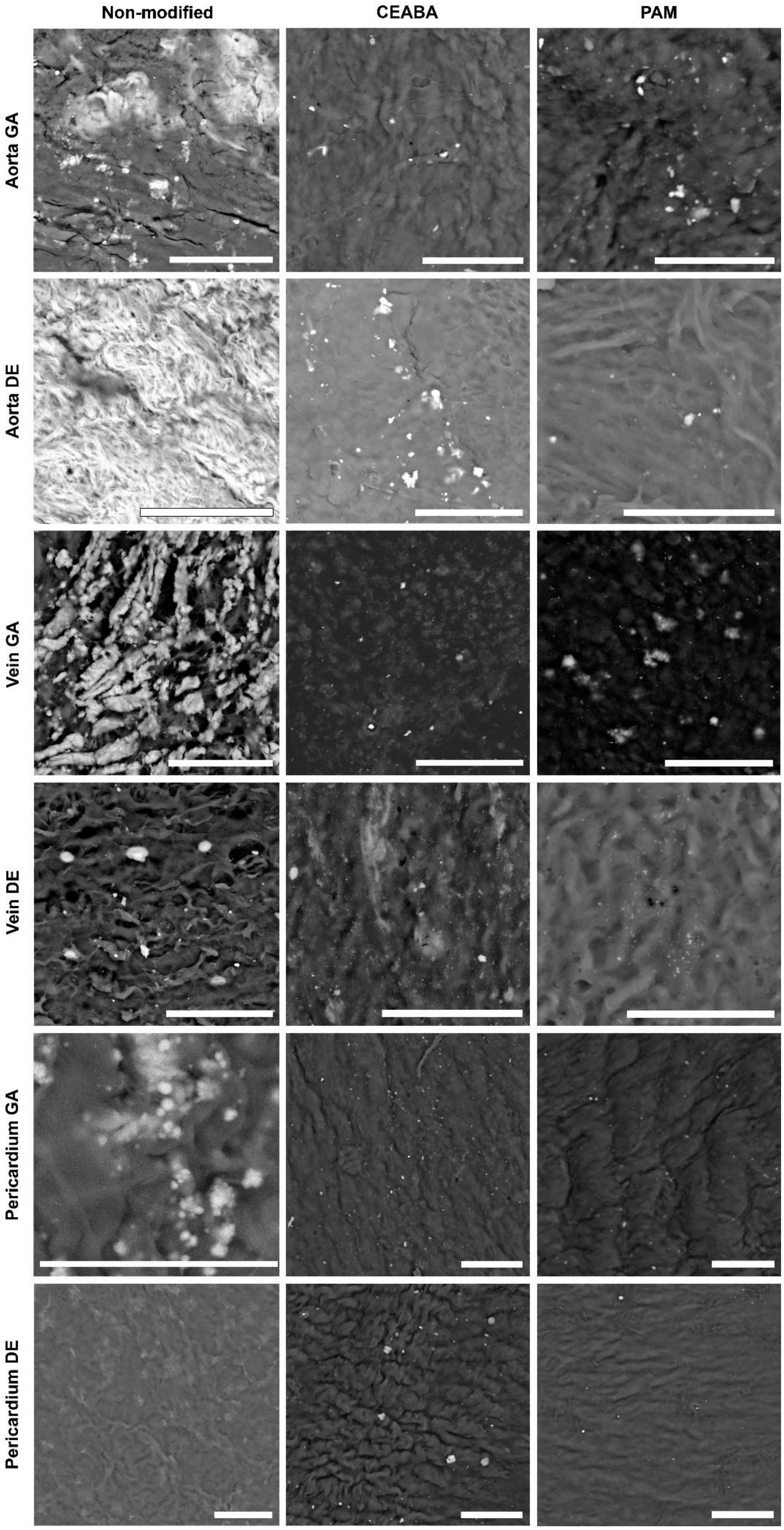

Figure S5. SEM (back-scattering electron) of the bioprosthetic material surfaces in 30 days after subcutaneous implantation in rats. Scale bars 50 μm.
